# RGMa Regulates Cortical Interneuron Migration and Differentiation

**DOI:** 10.1371/journal.pone.0081711

**Published:** 2013-11-27

**Authors:** Conor O'Leary, Stacey J. Cole, Michael Langford, Jayani Hewage, Amanda White, Helen M. Cooper

**Affiliations:** The University of Queensland, Queensland Brain Institute, Brisbane, Queensland, Australia; Institut de la Vision, France

## Abstract

The etiology of neuropsychiatric disorders, including schizophrenia and autism, has been linked to a failure to establish the intricate neural network comprising excitatory pyramidal and inhibitory interneurons during neocortex development. A large proportion of cortical inhibitory interneurons originate in the medial ganglionic eminence (MGE) of the ventral telencephalon and then migrate through the ventral subventricular zone, across the corticostriatal junction, into the embryonic cortex. Successful navigation of newborn interneurons through the complex environment of the ventral telencephalon is governed by spatiotemporally restricted deployment of both chemorepulsive and chemoattractive guidance cues which work in concert to create a migratory corridor. Despite the expanding list of interneuron guidance cues, cues responsible for preventing interneurons from re-entering the ventricular zone of the ganglionic eminences have not been well characterized. Here we provide evidence that the chemorepulsive axon guidance cue, RGMa (Repulsive Guidance Molecule a), may fulfill this function. The ventricular zone restricted expression of *RGMa* in the ganglionic eminences and the presence of its receptor, Neogenin, in the ventricular zone and on newborn and maturing MGE-derived interneurons implicates RGMa-Neogenin interactions in interneuron differentiation and migration. Using an *in vitro* approach, we show that RGMa promotes interneuron differentiation by potentiating neurite outgrowth. In addition, using *in vitro* explant and migration assays, we provide evidence that RGMa is a repulsive guidance cue for newborn interneurons migrating out of the ganglionic eminence ventricular zone. Intriguingly, the alternative Neogenin ligand, Netrin-1, had no effect on migration. However, we observed complete abrogation of RGMa-induced chemorepulsion when newborn interneurons were simultaneously exposed to RGMa and Netrin-1 gradients, suggesting a novel mechanism for the tight regulation of RGMa-guided interneuron migration. We propose that during peak neurogenesis, repulsive RGMa-Neogenin interactions drive interneurons into the migratory corridor and prevent re-entry into the ventricular zone of the ganglionic eminences.

## Introduction

The ability of the neocortex to perceive, process and respond to the continuous incoming stream of complex multi-modal information is dependent on the intricate neural network established between the excitatory pyramidal neurons and inhibitory interneurons. Disruption of this finely balanced neural network by perturbation of interneuron function has now been clearly linked to the etiology of neuropsychiatric disorders, including schizophrenia and autism [1]. Cortical inhibitory (GABAergic) interneurons make up approximately 20% of cortical neurons and originate in the ventral telencephalon [2]–[4]. The majority of cortical interneurons are born in the medial ganglionic eminence (MGE) which gives rise to the somatostatin and parvalbumin subpopulations [3], [4]. The remainder of the cortical interneuron subpopulations are generated by the caudal ganglionic eminence or the preoptic area [3]–[5]. Within the ventricular zone (VZ) of the MGE, radial progenitors undergo symmetric self-renewing divisions to maintain the progenitor pool. Alternatively, asymmetric divisions generate one new radial progenitor and either a neuron or an intermediate progenitor [6]. Intermediate progenitors undergo symmetric neurogenic divisions within the subventricular zone (SVZ) of the MGE giving rise to the majority of cortical interneurons. The identity of each interneuron subpopulation is predetermined by the spatially restricted expression of key transcription factors that specify neuronal morphology, neurotransmitter subtype and synaptic connectivity [7]–[11]. Young interneurons then migrate as clonal cohorts through the cortical migratory corridor within the SVZ, across the corticostriatal junction, into the developing cortex [2], [3], [12], [13]. Beginning at embryonic day 12 (E12), the first wave of migration targets the early preplate and intermediate zone in the dorsal telencephalon. From E14 to E16 the marginal zone, the subplate and the intermediate zone/SVZ boundary comprise the major migratory routes into the cortex [3], [14]–[16].

Successful navigation of newborn interneurons through the complex environment of the ventral forebrain is governed by spatiotemporally restricted deployment of both chemorepulsive and chemoattractive guidance cues that work in concert to regulate migration by creating a migratory corridor through the SVZ [3], [17], [18]. Not surprisingly, many of these cues are also responsible for axon guidance. MGE interneurons travel deep though the SVZ of the lateral ganglionic eminence (LGE) and reach the cortical plate without entering the striatum. Newborn interneurons express the semaphorin receptors, *neuropilin-1* and *neuropilin-2*, while the chemorepulsive semaphorins, *sema-3A* and *sema*-*3F*, are expressed in the striatum, thereby impeding migration into this region [19], [20]. Expression of the repulsive axon guidance receptor, *Robo-1*, also ensures exclusion from the striatum by modulating semaphorin-neuropilin signaling rather than by interacting with its Slit ligands [21], [22]. Similarly, the repulsive activity of striatal-localized ephrin-A3 also prevents interneurons from penetrating the striatal territory [23]. Concomitantly, chemokines act as cortically derived attractive cues to steer interneurons into the correct migratory stream [24], [25]. The long-range secreted cues, neuregulin and GDNF, also attract interneurons into the cortical plate [26], [27]. Additionally, a membrane-bound form of neuregulin is present on the corridor cells but not in the VZ of the ganglionic eminences, creating a permissive environment for migration within the corridor [26]. Neurotransmitters can also influence the migration of maturing interneurons as GABA, dopamine and glycine receptors are required for entry into the cortical plate [28]–[30]. Despite this expanding list of guidance cues, those responsible for preventing interneurons from re-entering the VZ are not well characterized. The repulsive interaction between EphA4 on interneurons within the SVZ and ephrin-A5, restricted to the VZ of the ganglionic eminences, prevents re-entry into the VZ [31]. Here we provide evidence that the chemorepulsive axon guidance cue, RGMa (Repulsive Guidance Molecule a), may also prevent interneurons from penetrating the VZ.

RGMa was first identified as the cue responsible for mapping temporal retinal axons onto the posterior region of the chick tectum [32]. Subsequently, the netrin receptor Neogenin (Neo) was identified as the guidance receptor mediating RGMa chemorepulsion [33]. Neo is now recognized as a bifunctional guidance receptor able to promote RGMa-mediated repulsion as well as chemoattraction in response to Netrin-1 [33]–[36]. RGMa is linked to the plasma membrane via a glycosylphosphatidylinositol (GPI) linkage but can also be cleaved to produce several soluble versions [37], [38]. Therefore it can act as both a membrane-bound, short-range guidance cue or a secreted, long-range cue. A detrimental role for RGMa has been demonstrated in the adult after CNS injury and stroke [39]–[42]. Additionally, central roles for RGMa and Neo are now emerging in a broad range of developmental processes, including neural tube formation [38], [43]–[45], neurogenesis in the embryonic and adult brain [46]–[48], cell adhesion in the early *Xenopus* gastrula [49] and endochondral bone formation [50]. More recently, RGMa-Neo interactions have also been implicated in pathological processes such as leukocyte chemotaxis during inflammation [51] and autoimmune multiple sclerosis [52].

Recently, a microdeletion within the *RGMa* locus has been linked to epilepsy and autistic behavior [53], thereby implicating it in cortical development. We have previously shown that *Neo* is expressed within the ganglionic eminences when interneuron differentiation and migration are at their peak [35], [47], [54], [55]. Furthermore, loss of Neo results in disruption of interneuron migration through the ventral forebrain [56]. In the current study, using an *in vitro* approach, we tested the hypothesis that RGMa is the relevant Neo ligand in the context of cortical interneuron differentiation and tangential migration through the SVZ of the ventral telencephalon. We present evidence that RGMa promotes neuronal differentiation by potentiating neurite outgrowth. We also show that RGMa is a repulsive guidance cue for newborn interneurons migrating away from the MGE.

## Results

### Coexpression of Neo and RGMa in the VZ of the Ganglionic Eminences

**Figure 1:**
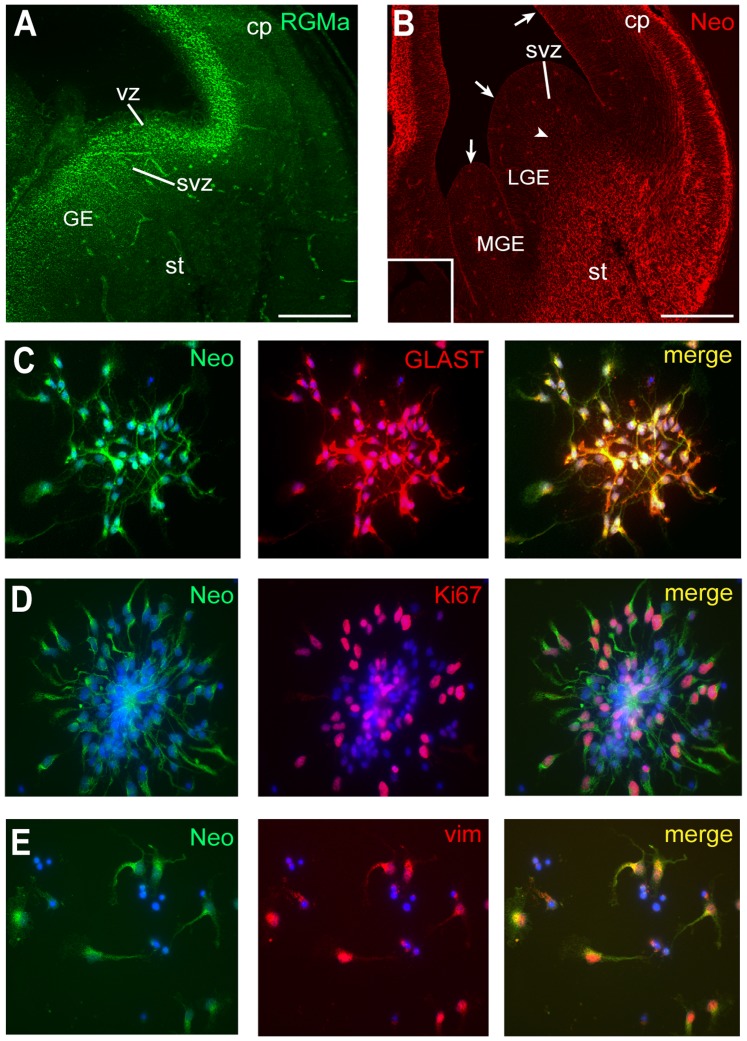
*RGMa* and *Neo* are expressed by radial progenitors in the VZ of the ganglionic eminences. (A) Coronal sections of E14.5 embryos show that RGMa was restricted to the VZ and SVZ in the ganglionic eminences. (B) Low levels of Neo were detected on radial progenitors in the LGE and MGE VZ (arrows). Newborn neurons within the migratory corridor were also Neo-positive (arrowhead). Neurons in the cortical plate (cp) and striatum (st) expressed high levels of *Neo*. No immunoreactivity was seen with an isotype-matched IgG control antibody (inset). Colabeling of E14.5 MGE VZ cells cultured for 2 days with antibodies to Neo (H-175, green), the radial progenitor marker, GLAST (C, red; merge, yellow), the cell cycle marker, Ki67 (D, red; merge, yellow), or the M-phase marker, phospho-vimentin55 (E, red; merge, yellow). GE, ganglionic eminence. Scale bars: A, 670 µm; B, 1.00 mm.

We initially examined the protein localization of RGMa in the E14.5 forebrain when interneuron production in the MGE and migration though the LGE into the dorsal telencephalon were at their peak. Strong RGMa immunoreactivity was seen in the VZ of the ganglionic eminences and extended into the SVZ (Fig. 1A). High levels of RGMa were also seen in the VZ of the dorsal telencephalon. However, *RGMa* was not expressed in the maturing neurons of the striatum or cortical plate. Thus, RGMa is appropriately positioned to influence the birth and migration of interneurons. Moreover, given that it is proteolytically cleaved into several soluble fragments [37], a chemotactic gradient is likely be established to guide newborn interneurons into the migratory corridor leading to the developing cortex.

We next examined the localization of the RGMa receptor, Neo, in the E14.5 forebrain. Low levels of Neo immunoreactivity were observed on the radial progenitors within the VZ of both the dorsal and ventral telencephalon, including the LGE and MGE, with the apical membrane of the progenitors exhibiting the highest level of Neo (Fig. 1B, arrows). To confirm that Neo was present on actively dividing progenitors, cells were isolated from the E14.5 MGE, cultured for 2 days and then colabeled with antibodies to Neo, the radial progenitor marker GLAST, the cell cycle marker Ki67, or the M-phase marker phospho-vimentin55. Neo was present on GLAST-positive radial progenitors (Fig. 1C) and on dividing progenitors coexpressing *Ki67* and *phospho-vimentin55* (Fig. 1D,E). In summary, *RGMa* and its receptor, *Neo*, are coexpressed on interneuron progenitors in the proliferative zone of the ganglionic eminences.

### *Neo* is Expressed by Interneurons Generated in the MGE

**Figure 2:**
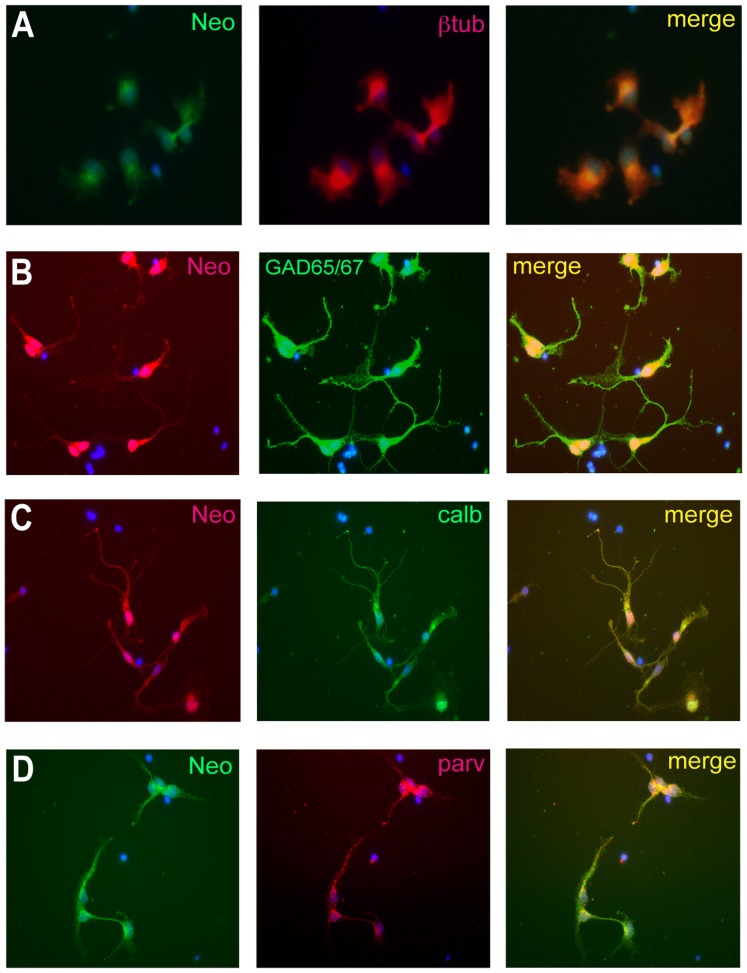
*Neo* is expressed on newborn cortical interneurons and the maturing calbindin and parvalbumin subpopulations. (A) 2 day cultures from the E14.5 MGE were immunolabeled with anti-Neo (H-175, green) and anti-βIII-tubulin (red; merge, yellow), a marker for newborn neurons. (B,C,D) MGE cells were isolated at E14.5, differentiated for 4 days and then colabeled with (B) anti-Neo (MAB1079, red) and anti-GAD65/67, a GABAergic interneuron marker (green; merge, yellow), (C) anti-Neo (MAB1079, red) and anti-calbindin (green; merge, yellow), or (D) anti-Neo (H-175, green) and anti-parvalbumin (red; merge, yellow).

Immunohistochemical analysis also showed that Neo was present on the soma of neurons in the cortical plate and striatum of the E14.5 forebrain (Fig. 1B). Brightly labeled individual cells were also found in the migratory corridor within the LGE (Fig. 1B, arrowhead), which are likely to be migrating newborn interneurons. To verify that *Neo* was expressed by newborn MGE-derived interneurons, cells were isolated from the E14.5 MGE, cultured for 2 days and then immunolabeled with an antibody specific for βIII-tubulin, a marker for newborn neurons. Neo was present on both the soma and emerging processes of the young βIII-tubulin-positive neurons (Fig. 2A). The MGE gives rise to calbindin and parvalbumin interneuron populations which leave the SVZ between E12 and E15 and migrate to the cortex via the LGE [14]–[16]. To further characterize *Neo* interneuron expression, MGE cells were isolated at E14.5, differentiated for 4 days and then immunostained for the general GABAergic interneuron marker, Glutamic Acid Decarboxylase (GAD65/67) or the interneuron subpopulation markers, calbindin and parvalbumin. Neo immunoreactivity was detected on maturing interneurons expressing each of these markers (Fig. 2B–D). Thus, Neo was present on newborn MGE-derived interneurons and continued to be expressed as they matured into the calbindin and parvalbumin subpopulations. Therefore, the spatiotemporal pattern of *Neo* expression suggests that it may be important in interneuron differentiation and migration.

### RGMa Promotes Interneuron Neurite Outgrowth but not Interneuron Production

**Figure 3:**
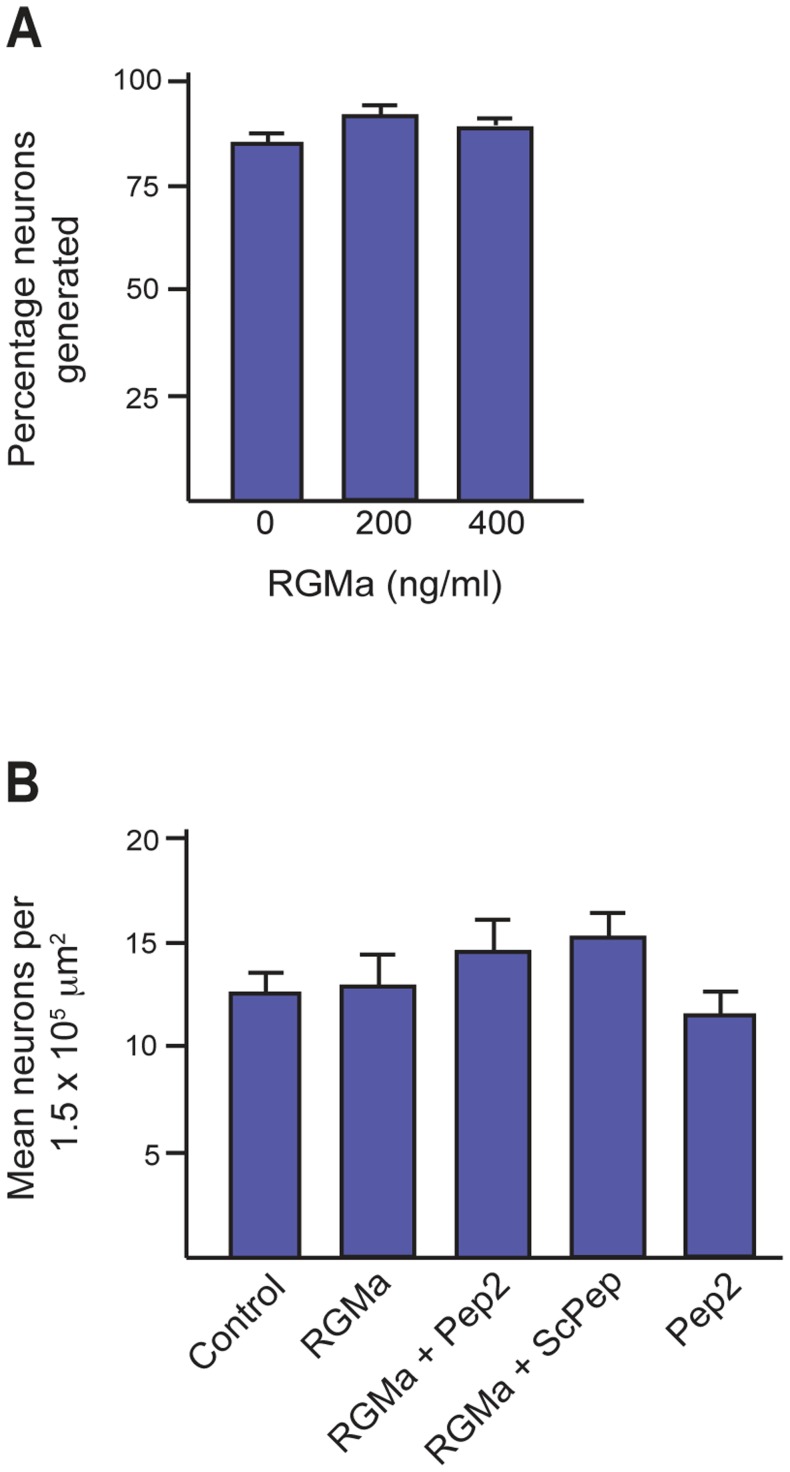
RGMa does not influence neurogenic divisions or interneuronal fate. MGE cells were isolated at E14.5 and differentiated for 4 days in recombinant RGMa. (A) No significant difference was observed in the percentage of neurons generated in the presence of 200 or 400 ng/ml RGMa or in the absence of RGMa. (B) Addition of the RGMa inhibitory peptide (Pep2, 10 µM) or scrambled control peptide (ScPep, 10 µM) had no significant effect on the number of neurons generated in the presence or absence of RGMa. Number of neurons counted per condition >520.

Interneuron progenitors divide asymmetrically to generate a new progenitor and a neuron, whereas symmetric neurogenic divisions give rise to two daughter neurons [6]. The expression of *RGMa* in the ganglionic eminence VZ suggested that it may play a role in the initial decision to take up a neuronal fate. To investigate this possibility the MGE was dissected from E14.5 embryos and cells dissociated and plated at clonal density in the presence or absence of RGMa (400 ng/ml). After 4 days cell fate was determined by labeling with anti-βIII-tubulin and the nuclear marker, DAPI. The production of interneurons was quantified and the number of βIII-tubulin-positive neurons expressed as a percentage of total cells (DAPI-positive). We found no significant difference in the number of neurons generated in the presence or absence of RGMa (Fig. 3A). As it was possible that RGMa produced by cells within the cultures had induced maximal differentiation in the absence of exogenous RGMa, we inhibited potential RGMa-Neo interactions by applying an RGMa peptide (Pep2, 10 µM) known to act as an RGMa antagonist [57]. Addition of this peptide without exogenous RGMa, or, in the presence of 400 ng/ml RGMa had no effect on the number of βIII-tubulin-positive neurons differentiating *in vitro* (Fig. 3B). Similarly, incubation with RGMa and the scrambled control peptide (ScPep, 10 µM) had no effect on interneuron differentiation. Therefore, RGMa does not initiate neuronal differentiation of MGE progenitors.

**Figure 4:**
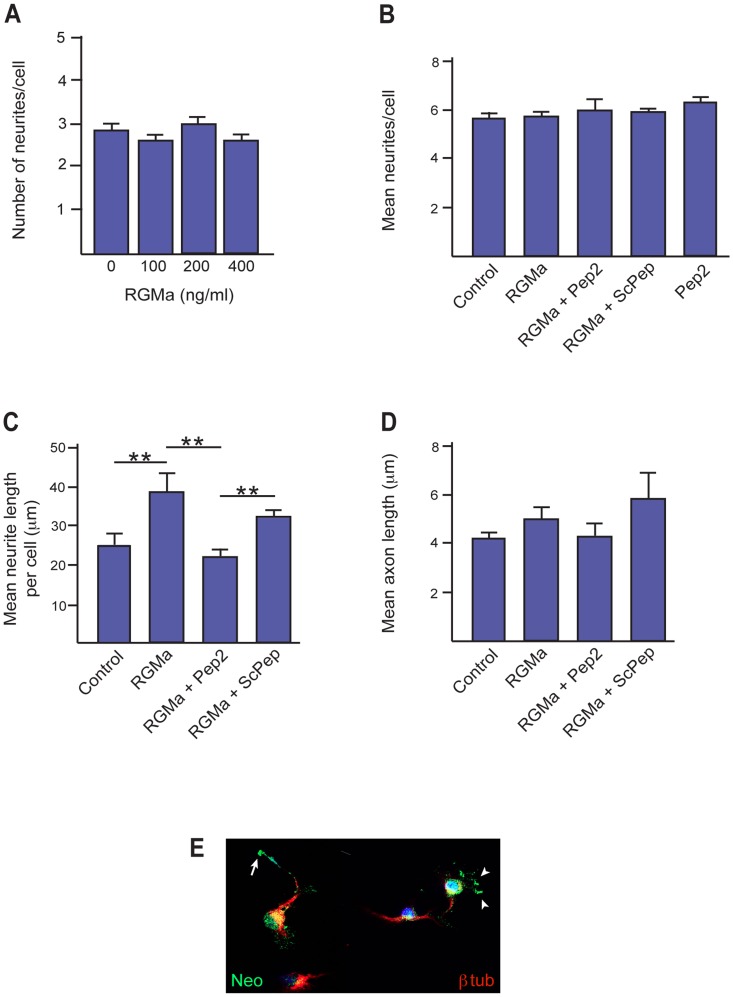
RGMa promotes neurite extension in differentiating interneurons. (A) E14.5 GE cells were differentiated for 4 days in the presence of 0–400 ng/ml RGMa and neurons labeled with anti-βIII-tubulin. (A) There was no significant difference in the total number of neurites elaborated per neuron in the presence of RGMa. (B) Addition of the inhibitory peptide Pep2 (10 µM), or control peptide ScPep (10 µM), had no significant effect in the presence (400 ng/ml) or absence of RGMa. Number of neurons counted per condition >520. (C) A significant increase in neurite length was observed in the presence of 400 ng/ml RGMa. RGMa-induced outgrowth was abrogated by Pep2 (10 µM) but not ScPep (10 µM). Number of neurons counted per condition >520. **p<0.01. (D) RGMa (400 ng/ml) did not enhance axon length (longest neurite). Number of axons counted per condition >90. (E) Colabeling of differentiating interneurons with anti-Neo (H-175, green) and anti-βIII-tubulin (red) showed Neo concentrated in the filopodia of neurites extending from the cell body (arrowheads) and in growth cone (arrow).

Neurite extension from the newborn neuron is a critical step in neuronal differentiation and maturation [60]. To determine if RGMa plays a role in this aspect of interneuron differentiation, E14.5 MGE cells were differentiated for 4 days in the presence of increasing concentrations of recombinant RGMa. The extent of neurite outgrowth was then assessed by determining the mean neurite number for each βIII-tubulin-positive cell. Fig. 4A shows that the addition of 100 to 400 ng/ml RGMa did not affect the total number of neurites produced per cell. Moreover, addition of the RGMa inhibitory peptide, Pep2 (10 µM), in the presence or absence of exogenous RGMa had no effect on neurite number (Fig. 4B), demonstrating that neurite induction was not influenced by endogenous RGMa activity within these cultures.

Many guidance cues also regulate neurite outgrowth. Therefore, we next investigated the ability of RGMa to modulate neurite growth by assessing the average length of neurites per cell. As RGMa acts as a chemorepulsive axon guidance cue, we predicted that the addition of RGMa would inhibit neurite extension. Unexpectedly, we observed a significant increase in neurite length in the presence of 400 ng/ml of RGMa (no ligand, 24.99±3.3 µm; RGMa, 38.6±5.2 µm; p = 0.008) (Fig. 4C). RGMa-induced outgrowth was abrogated in the presence of the inhibitory peptide (10 µM), whereas there was no significant difference between RGMa alone and RGMa+ScPep (RGMa, 38.6±5.2 µm; RGMa+Pep2, 21.97±1.9 µm; p = 0.001; RGMa+ScPep, 30.6±1.9 µm; p = 0.480) (Fig. 4C). In the above assay we assessed all neurites extending from each cell. This population comprised both the emerging axon and dendrites. We also analyzed the effect of RGMa specifically on axon growth by determining the length of the longest neurite on each cell. Fig. 4D shows that RGMa did not affect axon outgrowth. Therefore, RGMa promoted general neurite extension in MGE-derived interneurons, indicating that it is important for this facet of interneuron differentiation. To determine whether Neo was localized to the growth cones and filopodia of the extending neurites, 4-day MGE cultures were immunostained for Neo and βIII-tubulin. Neo was present on neurites projecting from the differentiating interneurons and was predominantly localized to the growth cones (Fig. 4E, arrow) and filopodia (Fig. 4E, arrowheads), suggesting that Neo is the RGMa receptor in the context of neurite outgrowth.

### RGMa and Netrin-1 Act Antagonistically to Guide Interneuron Migration

**Figure 5:**
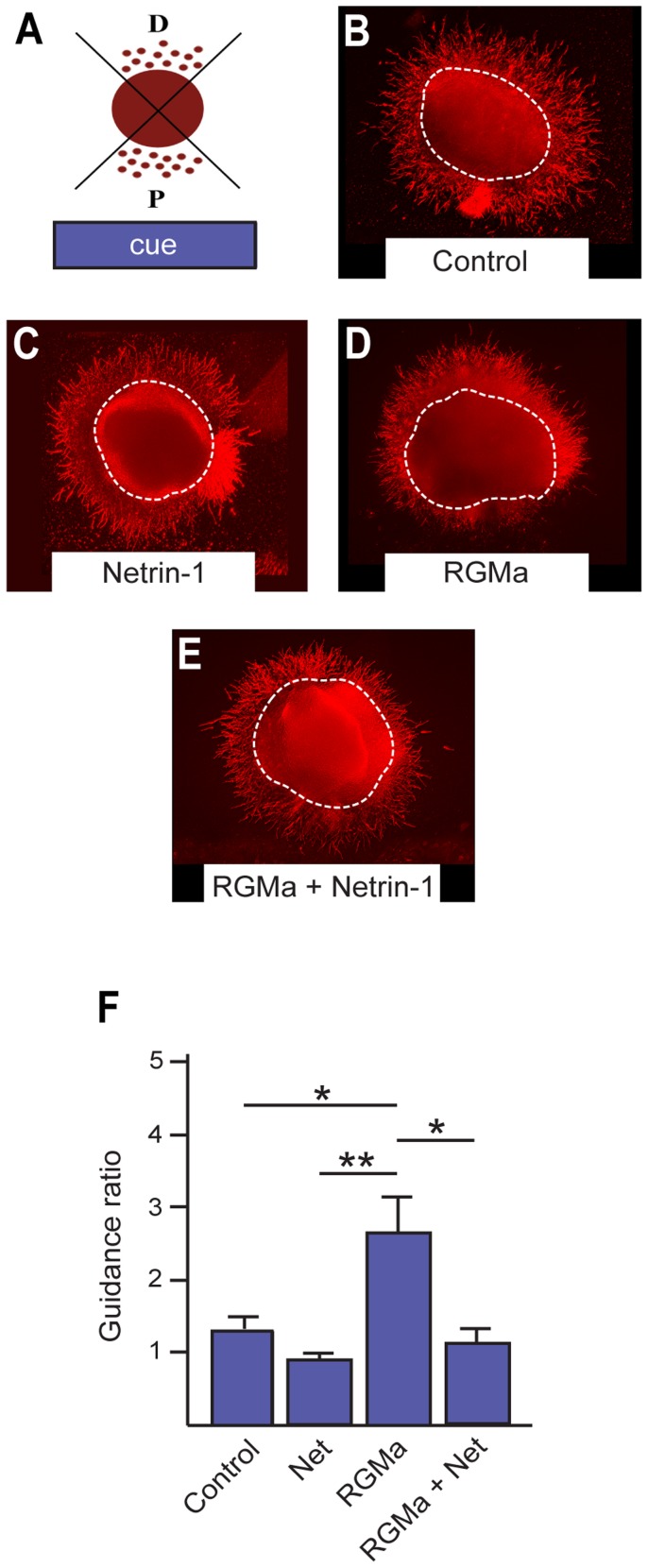
RGMa-mediated repulsion of newborn interneurons is suppressed by Netrin-1. (A) The extent of interneuron migration was calculated as the ratio of the distal to proximal area (guidance ratio) occupied by migrating cells. (B–E) Anti-βIII-tubulin immunolabeling (red) revealed the extent of interneuron migration out of E14.5 MGE VZ explants placed adjacent to agarose blocks containing control HEK293 cells (B), cells producing Netrin-1 (C), RGMa (D) or RGMa + Netrin-1 (E). Note the significant increase in neuron density on the distal side of the explant in the presence of RGMa (D). (E) Quantification of the guidance ratio for newborn interneuron migration out of the MGE explants in response to guidance cues. Dotted lines indicate the body of the explant. Control, n = 10; Netrin-1, n = 8; RGMa, n = 12; RGMa + Netrin-1, n = 7. *p<0.5, **p<0.01.

RGMa is known to induce a Neo-dependent chemorepulsive axon guidance response, whereas Netrin-1-Neo interactions are chemoattractive [33]–[36], [41]. The restricted localization of RGMa to the VZ of the ganglionic eminences and the expression of *Neo* on newborn interneurons suggested that RGMa-Neo interactions may repel newborn interneurons away from the VZ and along the migratory corridor leading to the cortex. In addition, *Netrin-1* is known to be expressed in both the E14.5 ganglionic eminence VZ and the striatum [56], [61], suggesting that Netrin-1-dependent guidance may also influence interneuron migration. To investigate the migration of interneurons in response to RGMa and Netrin-1 we performed *in vitro* explant assays in which MGE explants were dissected from the E14.5 forebrain and apposed to agarose blocks containing cells that produced RGMa, Netrin-1, or RGMa and Netrin-1 together (Fig. 5). Control blocks contained cells transfected with the empty expression vector. Explants were cocultured for 48 hrs and then stained with anti-βIII-tubulin to label neurons migrating from the explants. The extent of migration from the explant towards (proximal quadrant) or away from (distal quadrant) the ligand source was assessed by the guidance ratio. The guidance ratio was calculated by dividing the area occupied by neurons in the distal quadrant by the equivalent area in the proximal quadrant (Fig. 5A) [27]. Guidance ratios with values greater than 1 indicate repulsion, whereas ratios less than 1 indicate attraction.

When apposed to the control or Netrin-1 blocks the guidance ratio was close to 1 (Fig. 5B,C,F), indicating that there was no directed guidance of neurons away from (repulsion) or towards (attraction) the block. In contrast, the guidance ratio for explants exposed to the RGMa block was significantly greater than 1 (control, 1.30±0.20; RGMa, 2.65±0.48; p<0.05; Netrin-1, 0.87±1.3) (Fig. 5D,F), thereby revealing a strong chemorepulsive response to RGMa. Interestingly, RGMa-induced repulsion was abrogated when migrating interneurons simultaneously encountered RGMa and Netrin-1 within the same gradient (Fig. 5E,F). Under these conditions the guidance ratio returned to 1 (RGMa + Netrin-1, 1.10±0.25, p<0.05), indicating that Netrin-1 was acting to suppress RGMa chemorepulsive activity.

**Figure 6:**
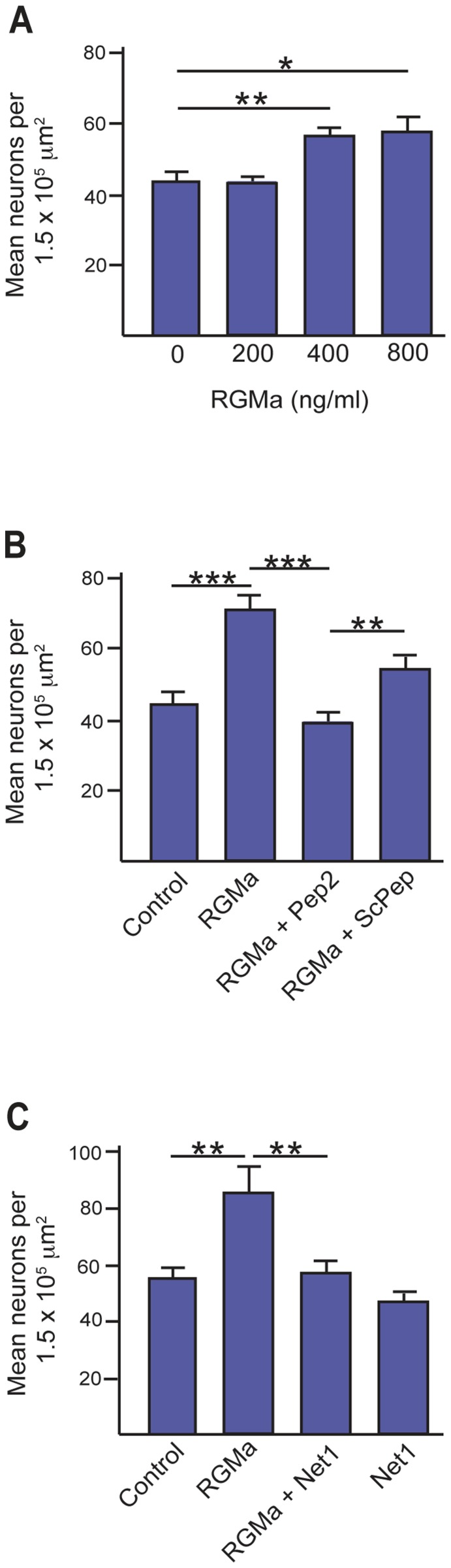
*In vitro* transwell assays confirm RGMa-mediated interneuron repulsion is inhibited by Netrin-1. (A) Interneuron migration into the lower chamber increased as the concentration of RGMa increased in the upper chamber (0 to 800 ng/ml RGMa). Number of neurons counted per condition >640. (B) RGMa (400 ng/ml) repulsion was abrogated when Pep2 (10 µM) but not the control peptide ScPep (10 µM) was added to the upper chamber containing RGMa. Number of neurons counted per condition >2,900.(C) Interneurons were exposed to equimolar concentrations of Netrin-1 (400 ng/ml) and RGMa (400 ng/ml), either alone or in combination. Netrin-1 fully inhibited RGMa-mediated interneuron repulsion when both cues were presented together. Number of neurons counted per condition >2,700. *p<0.5, **p<0.01, ***p<0.001.

To confirm that RGMa was acting as a chemorepulsive cue for migrating interneurons and that Netrin-1 suppressed this response, we developed an *in vitro* migration assay in which single-cell suspensions derived from the E14.5 ganglionic eminence were placed on the upper membranes of transwell chambers. Guidance cues were added to the upper chamber and the number of βIII-tubulin-positive neurons migrating into the lower chamber was assessed 24 hrs later. In agreement with the explant assay (Fig. 5), we observed increased interneuron migration into the lower chamber after addition of 400–800 ng/ml RGMa to the upper chamber (no RGMa, 43.6±2.8 cells; 400 ng/ml RGMa, 56.1±2.6 cells, p = 0.004; 800 ng/ml RGMa, 57.4±4.0 cells, p = 0.012) (Fig. 6A), indicating that RGMa was again acting as a repulsive guidance cue. Repulsion was abrogated when the inhibitory peptide (Pep2, 10 µM) was added to the upper chamber containing 400 ng/ml RGMa (RGMa, 70.65±4.2 cells; RGMa+Pep2, 38.9±2.8 cells; p = 0.0001) (Fig. 6B), thereby confirming the specificity of RGMa-mediated repulsion. Finally, to assess the ability of Netrin-1 to suppress RGMa-mediated repulsion, interneurons were exposed to equimolar concentrations of Netrin-1 (400 ng/ml) and RGMa (400 ng/ml), either alone or in combination. As seen in the explant assay (Fig. 5), Netrin-1 fully inhibited RGMa-mediated interneuron repulsion when both cues were presented together (no ligand, 55.2±3.8 cells; RGMa, 86.0±8.7 cells, p = 0.009; RGMa+Netrin-1, 56.9±4.1 cells, p = 0.831; Netrin-1, 47.2±2.7 cells, p = 0.183) (Fig. 6C), confirming that RGMa repulsion was suppressed by Netrin-1.

In summary, the data in Figs. 5 and 6 show that RGMa acts as an effective chemorepulsive guidance cue for newborn interneurons as they exit the ganglionic eminence VZ. In accordance with previous studies [17], Netrin-1 alone exhibits no interneuron guidance activity. However, unexpectedly, we found that Netrin-1 is able to inhibit RGMa-mediated chemorepulsion.

## Discussion

Large cohorts of newborn interneurons leave the MGE between E12 and E15 and migrate along the migratory corridor, before following predefined routes into the dorsal telencephalon [2], [3], [14]–[16]. The VZ/SVZ restricted expression of the chemorepulsive guidance cue *RGMa* and the presence of its receptor, Neo, on newborn MGE-derived GAD65/67-positive interneurons and maturing calbindin and parvalbumin interneuron subtypes implicated RGMa-Neo interactions in interneuron differentiation and migration. Here we present evidence to support this hypothesis. We show that RGMa potentiates neuronal differentiation by promoting neurite outgrowth. Despite the high level of *RGMa*-*Neo* coexpression in the VZ of the ganglionic eminences, we found that RGMa had no effect on the number of neurons generated, indicating that it did not influence the frequency of neurogenic divisions or neuronal fate. We also provide evidence that RGMa is a repulsive guidance cue for newborn interneurons migrating away from the MGE VZ. Conversely, in line with a previous report [17], Netrin-1 alone had no effect on migration, indicating that it is not a guidance cue for these interneurons. However, unexpectedly, we observed complete abrogation of RGMa-induced chemorepulsion when RGMa and Netrin-1 were encountered simultaneously by ganglionic eminence-derived interneurons. These intriguing findings reveal a novel mechanism for the tight regulation of RGMa-guided migration in the context of newborn cortical interneurons and suggests that RGMa and Netrin-1 may compete for receptor binding to control migration.

Throughout cortical neurogenesis Neo is localized to the VZ of the MGE and LGE and newborn interneurons (Figs. 1B, 2A). Furthermore, loss of Neo has been shown to inhibit interneuron migration in the SVZ [56]. DCC, a netrin receptor closely related to Neo, does not bind RGMa [33], [34] and thus cannot contribute to RGMa-mediated neurite outgrowth or interneuron migration. Together these observations strongly implicate Neo as the RGMa receptor in the ganglionic eminences.

### RGMa Enhances Interneuron Neurite Outgrowth

Unexpectedly, we found that RGMa promoted general neurite outgrowth in newborn MGE-derived interneurons but had no effect on the number of neurites elaborated or on axon growth. Previous studies have shown that RGMa inhibits neurite outgrowth of cortical neurons in a Neo-dependent manner [37], [62]. In addition, failure of corticospinal axons to regrow in spinal cord injury models has been attributed to the upregulation of *RGMa* expression [41], [42], [63], [64]. Moreover, RNA interference and RGMa antibodies suppress RGMa inhibitory activity after axonal injury and promote functional recovery [39]–[42]. Therefore, the ability to inhibit neurite outgrowth appears to be context-dependent. This is exemplified in the hippocampus where RGMa does not inhibit the outgrowth of entorhinal axons despite acting as a chemorepulsive cue for these axons [65]. In our study RGMa was applied to MGE cultures containing recently born interneurons or interneurons that had been generated within the culture as a result of progenitor neurogenic divisions. Therefore, RGMa was present at the beginning of neuronal differentiation when the nascent neurites were first specified. In contrast, RGMa-mediated outgrowth inhibition has been largely demonstrated in fully differentiated neuronal populations where axon projections were already well established.

Taken together these observations suggest that RGMa activity may be determined by the developmental context such as the differentiation state of the neuron or the neuronal subtype. RGMa has a variety of isoforms, including four GPI-linked, membrane bound forms and three soluble forms generated by proteolytic cleavage [37]. However, the biological activity of each isoform has yet to be established. Moreover, nothing is known about the tissue-specific or temporal patterns of RGMa cleavage. It is likely, however, that the relevant proteases will be differentially expressed by distinct cell types throughout development and into adulthood. Such complexity suggests that RGMa is likely to play a variety of roles in different developmental situations.

RGMa was initially identified as a chemorepulsive axon guidance cue. Thus, the promotion of neurite outgrowth appears contrary to what might be expected for a repulsive cue. However, it has been shown that Wnt5a can stimulate axonal outgrowth whilst simultaneously inducing a repulsive axon guidance response in cortical neurons [59], [66], [67]. The ability of Wnt5a to induce axonal outgrowth or repulsive guidance is determined by the complement of receptors on the growth cone [67]. In this system the interaction between Wnt5a and the repulsive guidance receptor, Ryk, is primarily responsible for axon outgrowth, whereas Ryk and the Frizzled receptor work together to promote chemorepulsive axon guidance by acting through a different arm of the Wnt/Ca^2+^ pathway. Similarly, RGMa's ability to stimulate neurite outgrowth may be dependent on the availability of Neo coreceptors such as Unc5 and CDO [62], [68]. Such context-dependent activity is best demonstrated by DCC which exhibits chemoattraction in a Netrin-1 gradient and repulsion when it forms a complex with Unc5 [69]. Interestingly, RGMa-induced repulsion requires a Neo-Unc5b receptor complex, at least in the context of cortical axon guidance [62].

In summary, the enhanced neurite outgrowth observed in differentiating cortical interneurons argues for a context-dependent response to RGMa-mediated receptor activation which may be further modulated by the RGMa isoform present in the local environment.

### RGMa is a Chemorepulsive Guidance Cue for Newborn Cortical Interneurons

Recent reports have revealed a role for RGMa in cell migration in the context of acute inflammation where RGMa chemorepulsion of lymphocytes and neutrophils was mediated by Neo [70]. Here we demonstrate for the first time that RGMa can also promote a significant repulsive response in a population of migrating interneurons (Figs. 5, 6). *Neo* is present on newborn βIII-tubulin-positive MGE-derived interneurons and continues to be expressed as these neurons mature into calbindin and parvalbumin subtypes (Figs. 1, 2). *RGMa* is expressed in the VZ of the ganglionic eminences and is thus well positioned to influence interneuronal migration along the migratory corridor. Therefore we propose that during peak neurogenesis, repulsive RGMa-Neo interactions drive the interneurons out of the MGE VZ into the migratory corridor. The presence of RGMa in the VZ of the ganglionic eminences further suggests that RGMa-mediated repulsion is required for the exclusion of migrating interneurons from the VZ along the entire extent of the migratory pathway in the ventral telencephalon.

It is well established that Netrin-1 plays important roles in cell migration and axon guidance [71], [72]. Moreover, VZ-derived Netrin-1 has been shown to attract matrix neurons into the striatum at later embryonic stages [73]. As seen in a previous study [17], Netrin-1 alone did not act as a guidance cue for E14.5 cortical interneurons. Surprisingly, however, Netrin-1 inhibited RGMa-mediated repulsive migration (Figs. 5, 6C), suggesting that the opposing actions of RGMa and Netrin-1 ensure migrating interneurons are restricted to the correct migratory pathway. An interplay between RGMa- and Netrin-1-mediated Neo activity has also been observed during *Xenopus* neural tube formation [43], [44]. In this developmental system *RGMa* and *Netrin-1* were expressed in complementary gradients across the neural plate and loss of either ligand or Neo produced delayed neural tube closure. RGMa-Neo interactions were required for the establishment of cell adhesion in the emerging neuroepithelium. In contrast, Netrin-1 did not regulate neuroepithelial morphogenesis, but may have acted to promote the migration of the neural plate cells towards the midline. Thus, as seen in interneuron migration, RGMa and Netrin-1 triggered differential responses upon binding to Neo.

How might Netrin-1 interfere with RGMa-mediated interneuron chemorepulsion at the molecular level? The binding site for RGMa has been mapped to the third and fourth fibronectin III domains of Neo [37]. Although the Netrin-1 binding site on Neo has yet to be identified, it is known that Netrin-1 binds the fourth and fifth fibronectin III domains of DCC [74], [75]. These Neo and DCC domains share 65 to 70% amino acid identity [34], [35], [76]. Thus, the Netrin-1 and RGMa binding sites on Neo are likely to be overlapping, suggesting that these ligands compete for Neo binding. In support of this postulate, preincubation with Netrin-1 suppresses RGMa-induced growth cone collapse in dorsal root ganglion axons [77].

On the basis of the above biochemical and functional data we propose the following model to explain the interplay between RGMa and Netrin-1 during interneuron migration in the ventral forebrain. The expression domains of *Netrin-1* are localized to the VZ of the ganglionic eminences and the striatum [56], [61] creating Netrin-1 gradients surrounding the migratory corridor. *RGMa*, on the other hand, is only expressed in the VZ and SVZ. As Neo has a higher affinity for RGMa than for Netrin-1 [33], it is likely that RGMa would be the dominant ligand within the VZ/SVZ where the secreted RGMa concentration is highest. In this situation RGMa would out-compete Netrin-1 for receptor binding, allowing RGMa-mediated repulsion to steer newborn interneurons away from the VZ/SVZ. Conversely, once in the migratory corridor, interneurons would experience low RGMa levels relative to the Netrin-1 gradient generated by the VZ and striatum. In this environment Netrin-1 would be predicted to out-compete RGMa for receptor binding, thereby suppressing RGMa-mediated repulsion and preventing migration into inappropriate territories such as the striatum.

### Conclusion

In this *in vitro* study we investigated the potential role of RGMa in several key phases of cortical interneuron development. We revealed that RGMa was able to promote interneuron differentiation by potentiating neurite outgrowth, but did not influence the initial decision to take up a neuronal fate. We further demonstrated that newborn MGE-derived interneurons exhibited a chemorepulsive response to RGMa. However, this response was abrogated when Netrin-1 was also present in the environment. Its localization in the VZ of the ganglionic eminences and on newborn interneurons strongly implicates Neo as the RGMa receptor mediating both neurite outgrowth and migration. This study now sets the scene for an in-depth *in vivo* analysis of RGMa-Neo function in cortical interneuron differentiation and migration.

## Materials and Methods

### E14.5 Forebrain Immunohistochemistry

The use of animals was approved by the Animal Ethics Committee of The University of Queensland in accordance with the guidelines stipulated by the National Health and Medical Research Council of Australia. E14.5 embryos were harvested and the brains immediately fixed in 4% paraformaldehyde (PFA) for 24 hrs at 4°C before embedding in low melting point agarose (Sigma-Aldrich Inc, USA). 30–50 µm sections were cut using a Leica VT1000s vibratome (Leica, Germany) and incubated in blocking solution (2% fetal calf serum, 2% goat serum, 0.2% Triton X-100 in PBS) for 1 hr at room temperature (RT) and then overnight at 4°C in primary antibody in blocking solution, followed by the secondary antibody for 1 hr at RT. *Antibodies*: goat anti-Neo (C20) (1∶100, Santa Cruz Biotechnology Inc., USA), rabbit anti-RGMa directed to the following sequence: RANAESPRRPAAASPSC. Isotype matched control antibodies: rat, mouse, rabbit IgG (Sigma). *Secondary Antibodies*: donkey anti-goat IgG or goat anti-rabbit IgG conjugated to AlexaFluor 488 or AlexaFluor 568 (1∶1000, Molecular Probes, Life Technologies, USA). Images were acquired on an Olympus IX81 inverted microscope using AnalySIS software (Olympus, Japan) or a Zeiss LSM510 Meta confocal microscope using LSM510 software (Oberkochem, Germany). Fluorescence levels and contrast were adjusted equally for all samples using Adobe Photoshop (Adobe Systems Inc., USA).

### MGE Cultures and Immunocytochemistry

Cells were dissociated from the MGE of E14.5 embryos and plated at 2×10^4^ cells/well onto poly-L-ornithine coated coverslips in 24 well plates in DMEM (Gibco, Life Technologies, USA) supplemented with 1% B-27 (Gibco), 1% N-2 (Gibco), 2 mM L-glutamine, 10 ng/ml β-fibroblast growth factor (Roche Diagnostics, Germany) and 100 U/ml penicillin and streptomycin. Cells were cultured for 2 or 4 days and then fixed in 4% PFA, blocked for 30 mins, and then incubated with primary antibody in blocking solution for 1 hr at RT and secondary antibody for 30 mins at RT. *Primary Antibodies*: mouse anti-Ki67 (1∶250, BD Pharmingen, USA), mouse anti-calbindin-D-28K (1∶800, Sigma), rabbit anti-GAD65/67 (1∶1000, Sigma), guinea pig anti-GLAST (1∶250, Millipore, USA), rabbit anti-Neo (H-175) (1∶250, Santa Cruz), rat anti-Neo (MAB1079, 1∶500, R&D Systems, USA), mouse anti-parvalbumin (1∶800, Sigma), rabbit anti-βIII-tubulin (1∶2000, Sigma), mouse anti-βIII-tubulin (1∶2000, Sigma), mouse anti-phosphorylated vimentin55 (1∶250, MBL, USA).

### Interneuron Differentiation Assays

Cells were dissociated from the ventral forebrain of E14.5 embryos and cultured as described above for 1 to 2 hrs before the addition of recombinant RGMa (R&D Systems). Cells were cultured for 4 days, replacing the RGMa-supplemented medium each day, after which cells were fixed and immunostained. *Cell differentiation assay*: Cells were immunolabeled with mouse anti-βIII-tubulin and DAPI (4′-6-diamidino-2-phenylindole, 1∶1000, Molecular Probes). The number of βIII-tubulin-positive neurons was expressed as a percentage of DAPI-positive cells. Alternatively, the mean number of βIII-tubulin-positive neurons was determined in the presence or absence of peptides (10 µM). RGMa inhibitory peptide (Pep2) (DFQAFRANAESPRR) corresponds to amino acids 309–322 in the C-terminus of rat RGMa [57]. This sequence is identical to the same region in mouse RGMa. Scrambled peptide sequence: SQRERPARFAAFDN. Peptides were synthesized by Genscript, USA. *Neurite Outgrowth Assay*: Cells were incubated in RGMa (100, 200, 400 ng/ml) in the presence or absence of peptides (10 µM) for 4 days. Cells were fixed using 4% PFA and immunostained with anti-βIII-tubulin and DAPI. Coverslips were mounted on slides using Prolong Gold (Molecular Probes) and imaged on a Zeiss Axio Imager microscope. Images were analyzed using the ImageJ (National Institute of Health, US), NeuriteQuant plugin [58]. The longest neurite of each cell was quantified using the NeuronJ plugin.

For all analyses 15 fields were counted per animal, n = 3 animals. Statistical significance was determined by the Kruskal-Wallis test followed by the Mann Whitney test. The level of significance was considered to be *p*<0.05 for all statistical analyses. Data are presented ± the standard error of the mean (sem).

### MGE Explant Assay

HEK293 cells were transfected with cDNAs encoding the RGMa extracellular domain cloned into the pShooter, pEF-myc-cyto mammalian expression vector (Invitrogen, Life Technologies, USA), pBK-CMV-Netrin-1-FLAG, a kind gift from Prof Andreas Püschel (Institut für Molekulare Zellbiologie, Münster) or the pEF-myc-cyto vector as the control using Fugene 6 (Roche Diagnostics) according to the manufacturer's instructions. To determine transfection efficiency, each construct was transfected at a ratio of 4∶1 with pEF-myc-cyto-GFP (Invitrogen). Explant assays were carried out as previously described [59]. Briefly, 24 hrs after transfection cells were resuspended in 20 µl of medium and mixed with 80 µl of 2% (w/v) low melting point agarose in DMEM/F12 (Gibco). MGE explants were dissected from E14.5 ventral forebrain and placed in 25 µl of liquid collagen (Millipore) at a distance of approximately 100 µm from the ligand-expressing cell blocks (∼300 µm^2^). Explant cocultures were incubated for 48 hrs at 37°C and then fixed in 4% PFA, blocked for 1 hr and then incubated overnight at 4°C with mouse anti-βIII-tubulin (1∶2000) followed by AlexaFluor 568 goat anti-mouse antibody (1∶1000, 1 hr, RT) and DAPI (1∶1000). Explants were imaged on an IX50 inverted fluorescence microscope (Olympus). Using AnalySIS software (Olympus) optical Z-slices through the explants were acquired and then stacked and merged to generate a single image. The extent of migration was determined by calculating the area occupied by migrating βIII-tubulin-positive cells in the region proximal or distal to the explant. The migratory response was expressed as the ratio (i.e. the guidance ratio) of the distal area to proximal area ± sem and statistical significance determined by one-way ANOVA followed by the Bonferroni's *post hoc* test.

### Transwell Migration Assay

Cells were dissociated from the ventral forebrain of E14.5 embryos and cultured as described above. Transwell membrane inserts (8 µm pore size, Falcon, BD Biosciences, USA) were placed in 24 well plates. A total of 200 µl of media containing 2×10^5^ cells was placed into the upper chamber (lower chamber; 500 µl media). RGMa, Netrin-1 and peptides were also added to the upper chamber. Twenty hrs post seeding the cells that had migrated into the lower chamber were fixed with 4% PFA and immunostained with anti-βIII-tubulin and DAPI and imaged on a Zeiss Axio Imager microscope. Images were analyzed using the ImageJ, NeuriteQuant plugin. The mean number ± sem of βIII-tubulin-positive neurons was determined over 15 fields per animal, n = 5 animals (Fig. 6A, B), n = 4 (Fig. 6C). Statistical significance was determined by the Kruskal-Wallis test followed by the Mann Whitney test.
